# Electrically controlled cloud of bulk nanobubbles in water solutions

**DOI:** 10.1371/journal.pone.0181727

**Published:** 2017-07-20

**Authors:** Alexander V. Postnikov, Ilia V. Uvarov, Mikhail V. Lokhanin, Vitaly B. Svetovoy

**Affiliations:** 1 Yaroslavl Branch of the Institute of Physics and Technology, Russian Academy of Sciencies, Yaroslavl, Russia; 2 Department of Physics, P. G. Demidov Yaroslavl State University, Yaroslavl, Russia; 3 Zernike Institute for Advanced Materials, University of Groningen, Groningen, The Netherlands; Massachusetts Institute of Technology, UNITED STATES

## Abstract

Using different experimental techniques we visualize a cloud of gas in water that is produced electrochemically by the alternating polarity process. Liquid enriched with gas does not contain bubbles strongly scattering visible light but its refractive index changes significantly near the electrodes. The change of the refractive index is a collective effect of bulk nanobubbles with a diameter smaller than 200 nm. Any alternative explanation fails to explain the magnitude of the effect. Spatial structure of the cloud is investigated with the optical lever method. Its dynamics is visualised observing optical distortion of the electrode images or using differential interference contrast method. The cloud covers concentric electrodes, in a steady state it is roughly hemispherical with a size two times larger than the size of the electrode structure. When the electrical pulses are switched off the cloud disappears in less than one second. The total concentration of gases can reach very high value estimated as 3.5 × 10^20^ cm^−3^ that corresponds to an effective supersaturation of 500 and 150 for hydrogen and oxygen, respectively.

## Introduction

Nanobubbles (NBs) are nanoscopic gaseous domains than can exist on solid surfaces or in the bulk of liquids. They attracted significant attention in the last decade [1–3] due to their long-time stability and high potential for applications. The NBs can be applied for nanocsopic cleaning [4–7], for control of boundary slip in microfluidics [8, 9], for wastewater treatment [10, 11], for heterocoagulation [12, 13], and for medical applications [14, 15]. An extensive literature exists on the surface NBs (see, for example, [2]), which were observed using different experimental methods. On the contrary, the bulk NBs are investigated much less.

Ohgaki *et al*. [16] produced the bulk NBs mechanically with a rotary pump reaching a rather high effective supersaturation (36 for nitrogen) and related reduction of the liquid density. The effective supersaturation counts the gas dissolved in the liquid so as the gas collected in small bubbles. The bubbles were visualized by scanning electron microscopy from freeze-fracture replicas. Detailed size distribution of oxygen NBs was determined by dynamic light scattering (DLS) by Ushikubo *et al*. [17] for smaller effective supersaturation 4–5. Bunkin *et al*. [18] observed NBs in sodium chloride solutions by DLS, phase microscopy, and polarimetric scatterometry and have been able to distinct latex particles from NBs. Yurchenko *et al*. [19] proposed that charges on the gas-water interface are responsible for exceptional stability of the NBs. In a series of papers by Kikuchi *et al*. [20–24] hydrogen and oxygen NBs were produced by the electrochemical process and investigated for a relatively small supersaturation of about 2. A cloud of O_2_ NBs around hydrophilic particles was observed with synchrotron technique by Pan *et al*. [25]. Although these bubbles are the surface NBs, it is the first time when a localized cloud of NBs is mentioned.

Extremely high effective supersaturation ∼ 1000 was reached in alternating polarity (AP) electrolysis of water (see [26] for a review) when polarity of electrodes is interchanged with a frequency of the order of 100 kHz. The reason for this level of supersaturation is much larger Faraday current density *j*_*F*_ ∼ 100 A/cm^2^ than that for normal electrolysis performed by DC voltage (*j*_*F*_ < 1 A/cm^2^ [27]). High supersaturation helps to overcome the nucleation barrier resulting in homogeneous nucleation of the bubbles on the time scale shorter than 10 *μ*s [28]. Only NBs are formed because no scattering of visible light is observed but a significant gas concentration was confirmed by different methods [29, 30]. It has to be stressed that high effective supersaturation can be supported due to high Laplace pressure in NBs. Moreover, these bubbles must be bulk NBs otherwise all the electrodes would be covered with gas and the current would stop. Because of fast change of polarity the high supersaturation with both hydrogen and oxygen gases exists above the same electrode. In this situation bubbles containing hydrogen, oxygen, or mixture of gases can be formed. The NBs produced in this way show very fast dynamics in comparison with the mechanical or normal electrolysis methods mentioned above.

The reason for the fast dynamics is not different physical properties of the bubbles but rather ability of the gases to interact. A significant part of the gases disappears in phase with the change of polarity [29, 30] as oscillations of the refractive index show. This observation was explained as spontaneous reaction between H_2_ and O_2_ gases inside of NBs. Normally, the gases cannot interact spontaneously at room temperature but in the NBs, where the surface-to-volume ratio is large, some surface channels for the reaction can be opened [31]. Generation of free radicals was already observed in water containing shrinking microbubbles filled with air, oxygen, or ozone in absence of external dynamic stimuli [32, 33].

The NBs containing only hydrogen or only oxygen are collected in the system. They produce overpressure in a microchamber closed with a flexible membrane. The overpressure was measured using the calibrated membrane deflection [30]. When the electrochemical current is switched off the overpressure relaxes in a few hundreds of microseconds suggesting that NBs containing H_2_ merge with those containing O_2_ and disappear in the reaction. If the overpressure becomes large (comparable with 1 bar), short-lived microbubles (MBs) visualized with a stroboscope appear in the chamber [34]. They live just a few microseconds and disappear with a significant energy release. In an open system these MBs were accompanied by clicking sounds and their dynamics was investigated with a fast camera [35]. These phenomena are well described by the combustion of gases inside of MBs. To produce a MB with a stoichiometric mixture of gases many NBs have to coalesce. For this the NBs have to be packed so densely that they practically touch each other. It was assumed in [34, 35] that such a state of liquid densely packed with NBs exists but it was not observed directly.

In this paper we investigate a state of liquid highly supersaturated with hydrogen and oxygen gases. With different experimental techniques we probe a cloud of gas above the electrodes and demonstrate that it can be controlled by electrical means. We argue that the gas in the cloud exists in the form of NBs.

## Experimental section

Sodium sulfate solution in Milli-Q water (15 g of Na_2_SO_4_ per 100 g H_2_O) is used as the electrolyte. In contrast with previous works where platinum [34] or copper [35] electrodes were used, in this experiment we sputter the electrodes containing three layers on an oxidized Si-wafer. The electrodes consist of a 10 nm Ti adhesion layer, 500 nm Al layer, and 100 nm Ti layer on top of the “sandwich”. Such electrodes can be used in the electrolysis for a long time without significant reduction of their properties.

The electrodes were patterned using a standard lithography (photoresist Microposit S1813, thickness 1.5 *μ*m). Circular shape of the electrodes provides a better localisation of the gas produced by the electrochemical process. The internal electrode has a diameter of 300 *μ*m and the external diameter of the structure is 700 *μ*m. The contact lines originally insulated by the photoresist are covered additionally by a compound Omnivisc 1050 to provide better chemical resistivity.

Square voltage pulses of alternating polarity are applied to the electrodes from an external generator. One electrode is grounded while the other one is at a positive or negative potential with respect to ground. The frequencies of the pulses in the experiments are *f* = 100, 200, and 400 kHz. We use continuously generated pulses, continuous pulses modulated by a sawtooth function, and series consisting of a fixed number of pulses. All electrical signals are recorded by a PicoScope 5000.

**Optical distortion**. A sample with the electrodes is fixed in a Petri dish and covered with 2–3 mm of the electrolyte. To observe optical distortion of the electrodes produced by the cloud (see S1 Video) we apply to the electrodes the pulses with an amplitude of *U* = 10 V and a frequency of *f* = 200 kHz. The video shows 3 series of pulses, each series contains 800 000 periods (one positive plus one negative pulses). Each series is triggered manually. The electrodes were observed in a microscope Mitutoyo FS70 (objective 10X EO M Plan Apo, long working distance, infinity corrected, numerical aperture 0.28, focus distance 20 mm, working distance 33.5 mm) with a mounted camera Nikon 1 J4 to record videos at a frame rate of 60 fps. A similar video was made with a thin (150 *μ*m) glass floating above the electrodes to avoid possible surface buckling.

**Fig 1:**
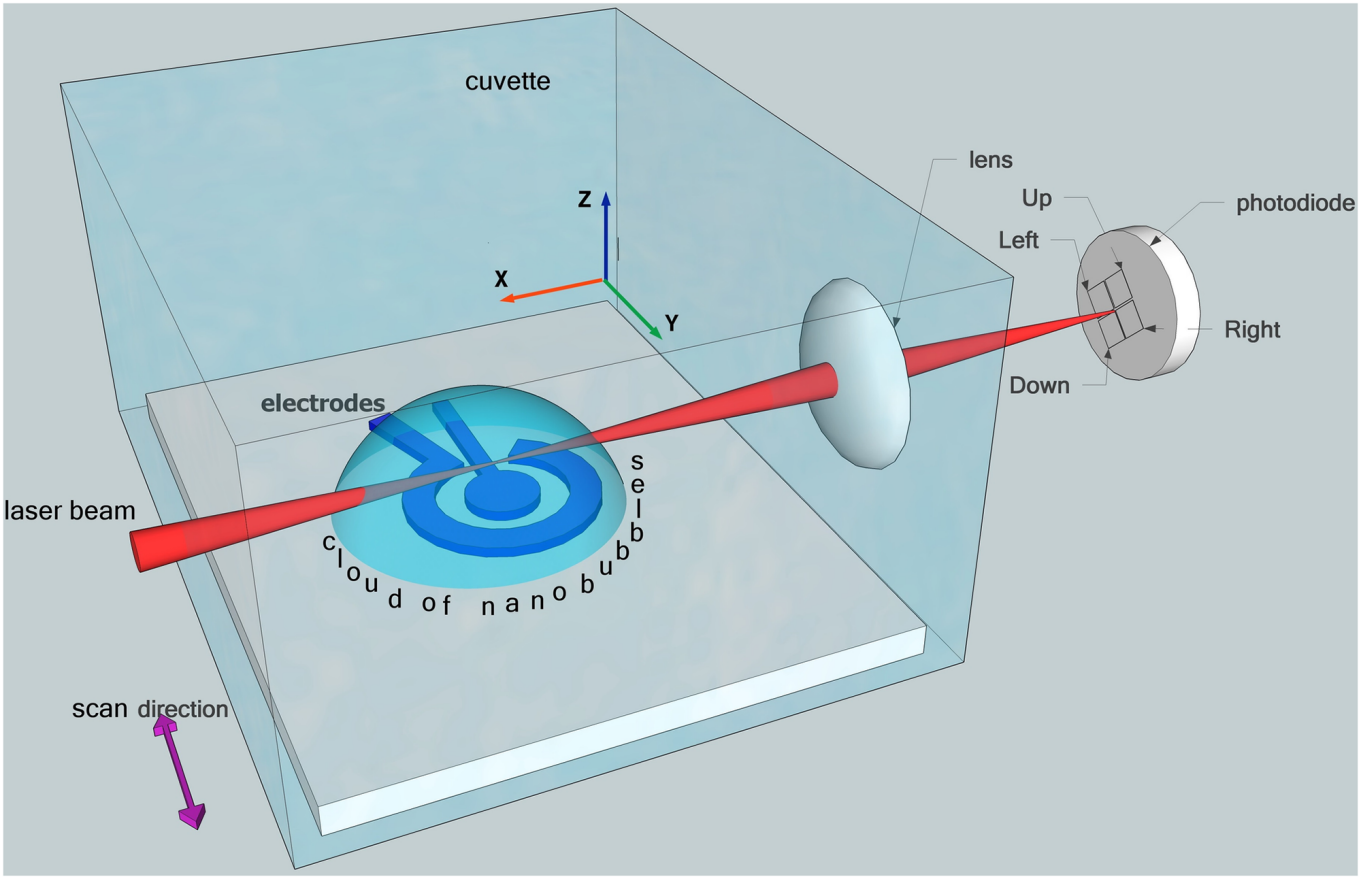
Scheme of the optical lever experiment. A transparent cuvette is mounted on a 3D stage and filled with the electrolyte. The sample with the electrodes is fixed at the bottom of the cuvette. A laser beam parallel to the sample surface is focused in the neighborhood of the electrodes. Deflection of the beam due to presence of the gas-enriched cloud is picked up by a four-section photodiode.

**Optical lever**. Optical lever method allows investigation of a space distribution of the gas cloud around the electrodes. The scheme of measurements is presented in Fig 1. The sample is fixed at the bottom of a specially made transparent cuvette with dimensions of 2 × 3 × 3 cm^3^. A laser beam (λ = 632 nm) passes through the cuvette parallel to the sample surface and is focused in the liquid volume. If the beam passes through the cloud, it is deflected due to the change of the refractive index of the liquid enriched with the gas. This deflection is measured by a four-section photodiode (PD). The cuvette with the sample can be moved independently along all three axis. By moving the cuvette along the laser beam (*X* axis) one can align the beam waist above the central electrode. Choosing the vertical position of the cuvette (*Z* axis) we can probe the cloud on different distances from the sample. A minimal vertical distance of 500 *μ*m between the focal spot and the sample is limited by spurious reflections. The *Z* coordinate can be chosen with an accuracy of 2 *μ*m within the range of 2 mm.

When the cuvette moves in *Y* direction, the beam waist scans above the electrode structure. The deflection of the beam is an integral effect of the change of the refractive index along the beam but the main contribution comes from the region nearby the beam waist. The beam passed through the cuvette is collected by a focusing lens on the PD. Scanning in the *Y* direction is realized by an electrodynamic driver, which gives smooth, well controllable, and repeatable motion.

**Differential interference contrast**. Differential interference contrast (DIC) microscopy uses [36] two mutually coherent orthogonally polarized beams, which are slightly displaced spatially at the sample plane. When the beams are combined an interference pattern depends on the optical path difference in the direction of the displacement. Therefore, DIC converts the gradient in the optical path into the amplitude difference that can significantly improve contrast. This method is appropriate for visualisation of the gas distribution in dynamics.

The AP voltage pulses were modulated by the sawtooth signal with a period of 5 s to observe the dynamics of the gas distribution. In this case the concentration of the gas increases, reaches a maximum, and decreases again. The interference pattern is observed in white incoherent light with a Nomarski objective 10x. The displacement of the two orthogonally polarized images was 30 *μ*m. The phase difference between the two images is encoded in the colormap (period λ/2). Since DIC is sensitive to only one direction of the phase gradient, the images were obtained for two orthogonal orientations of a splitting prism in the Nomarski objective.

**Vibrometer**. Short-time dynamics of the cloud can be visualised with a laser Doppler vibrometer, which measures the rate of change of the phase difference between the beam passed through the liquid and reflected from the sample and the reference beam reflected from a reference mirror within the instrument. We use a Polytec MSA-400 instrument. The laser beam (λ = 632 nm) with a spot size of ∼ 5 *μ*m is focused on the sample. A series of pulses are applied to the sample and the voltage, current, and vibrometer signal are recorded synchronously. We used different number of pulses ∼ 1000 and the beam is focused in different points on the structure to probe the gas concentration locally.

It has to be noted that interpretation of the vibrometer data is not straightforward and has to be taken with care. This is because not only change in the refractive index but also movement of the liquid both give contribution to the signal. On the time scale > 1 ms it is impossible to separate the two contributions.

## Results

Application of a constant potential to the electrodes results in the formation of titanium oxide on the positive electrode, which reduces the current and the electrochemical process stops. The oxide gives gold or copper-like color to the anode that is explained by the light interference rather than stoichiometric defects in the oxide [37]. Application of the AP pulses revives the electrochemical process since cathode and anode interchange with a high frequency.

**Fig 2:**
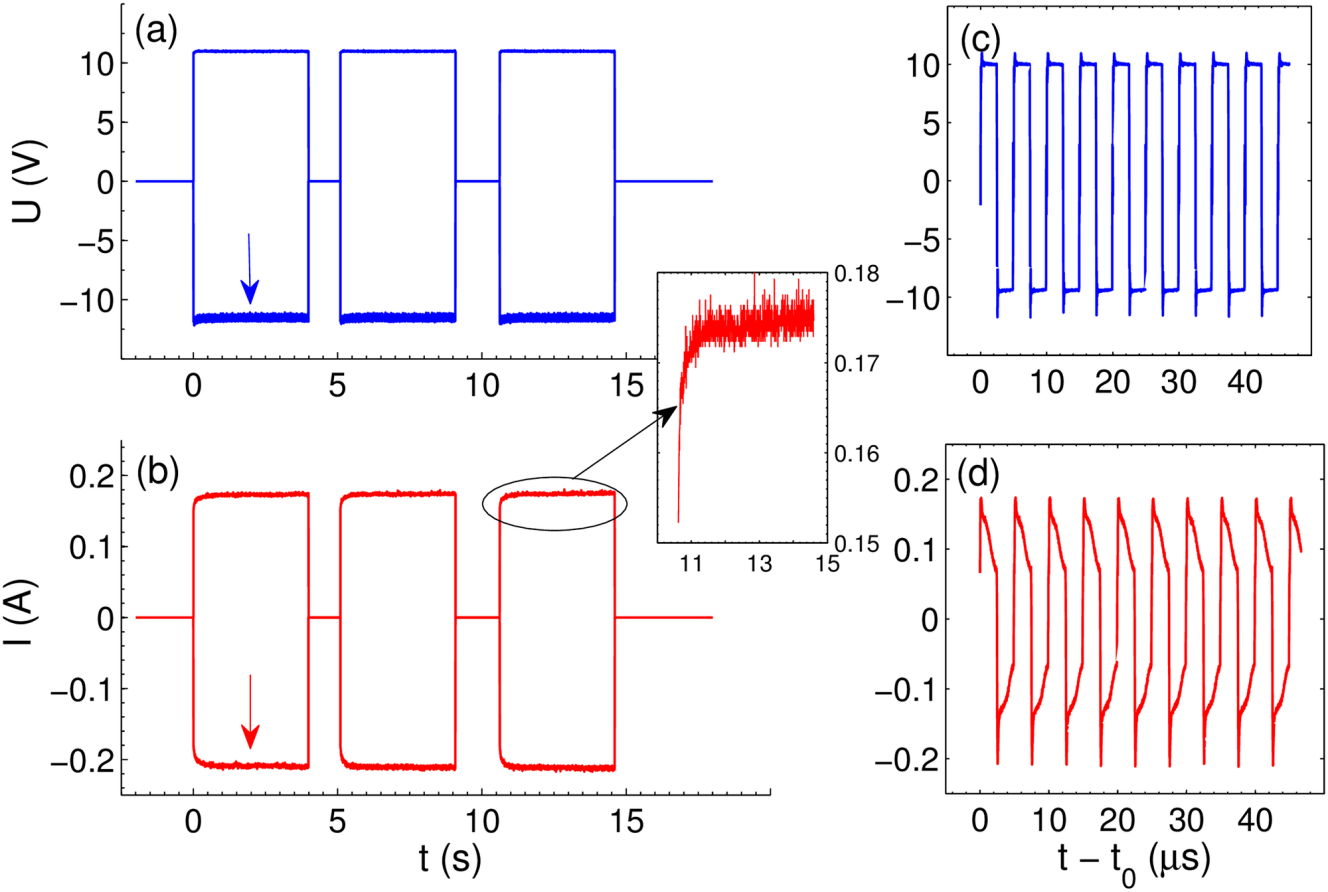
Applied voltage pulses and current response at *U* = 10 V and *f* = 200 kHz. (a) Three series of rectangular voltage pulses applied to the electrodes. Only enveloping line is shown. (b) Enveloping line for the current response. The inset shows the increase of the current amplitude with time for the third series of pulses. (c) Time resolved separate voltage pulses near *t*_0_ = 2 s (indicated by the blue arrow). (d) Time resolved current pulses near *t*_0_ = 2 s (indicated by the red arrow).

The AP voltage pulses and the corresponding current response are shown in Fig 2 for *U* = 10 V and *f* = 200 kHz. In previous investigations of the AP regime, where Pt, Au, Cu, or W electrodes were used, each current pulse was well fitted by the time dependence [28] *I*(*t*) = *I*_*F*_ + *I*_1_*e*^ − *t*/*τ*^, where *I*_*F*_ is the Faraday current and the second term describes the charging-discharging effects with a relaxation time *τ*. For Ti/Al electrodes this is not exactly the case as one can see in Fig 2(d). The three parametric fit is not good probably due to the formation and dissolution of titanium oxide, which also contributes to the current. Roughly *I*_*F*_ is estimated as 60 mA.

All the experimental methods described in this paper visualise the change in the refractive index *n* around the electrodes. Although we are not able to observe individual NBs due to fast dynamics, it is possible to make a definite statement that the change in *n* is induced by the gas presenting in liquid in the form of the NBs. This statement is based on the following experimental facts: (i) observed *n* is always smaller than the refractive index of the solution *n*_*s*_; (ii) the difference *n*_*s*_ − *n* is so large (up to 0.26) that it can be explained only by a significant amount of gas densely packed in NBs; (iii) absence of scattering of the visible light shows that the gas is collected in nanoobjects with a size smaller than 200 nm; (iv) the local temperature near the electrodes increases not more than 6.5°C that excludes vapor as a possible origin of the refractive index change. The data supporting these facts are discussed below.

To relate the change in the refractive index to a volume fraction of inclusions *f*_*i*_ (gas in our case) we are using the Bruggeman effective medium approximation (see, for example, [38]). It deals with the dielectric constants of the inclusions *ε*_*i*_, of the solution *ε*_*s*_, and of the effective medium *ε*, which are related by the equation

$$\begin{array}{c} f_{i}\frac{\varepsilon_{i}-\varepsilon }{\varepsilon_{i}+2\varepsilon }+\left(1-f_{i}\right)\frac{\varepsilon_{s}-\varepsilon }{\varepsilon_{s}+2\varepsilon }=0. \end{array}$$ (1)

The refractive index is expressed via the dielectric constant as $n=\sqrt{\varepsilon }$. In the visible light we can take for 1 mol/L Na_2_SO_4_ solution *n*_*s*_ ≈ 1.35. If we consider gas as the inclusions, then *n*_*i*_ = 1 and an approximate solution of Eq (1) is

$$\begin{array}{c} n_{s}-n\approx 0.352f_{g}. \end{array}$$ (2)

It holds true with a precision of 10^−3^ not only for small volume fractions of gas but for any *f*_*g*_.

### Optical distortion experiment

**Fig 3:**
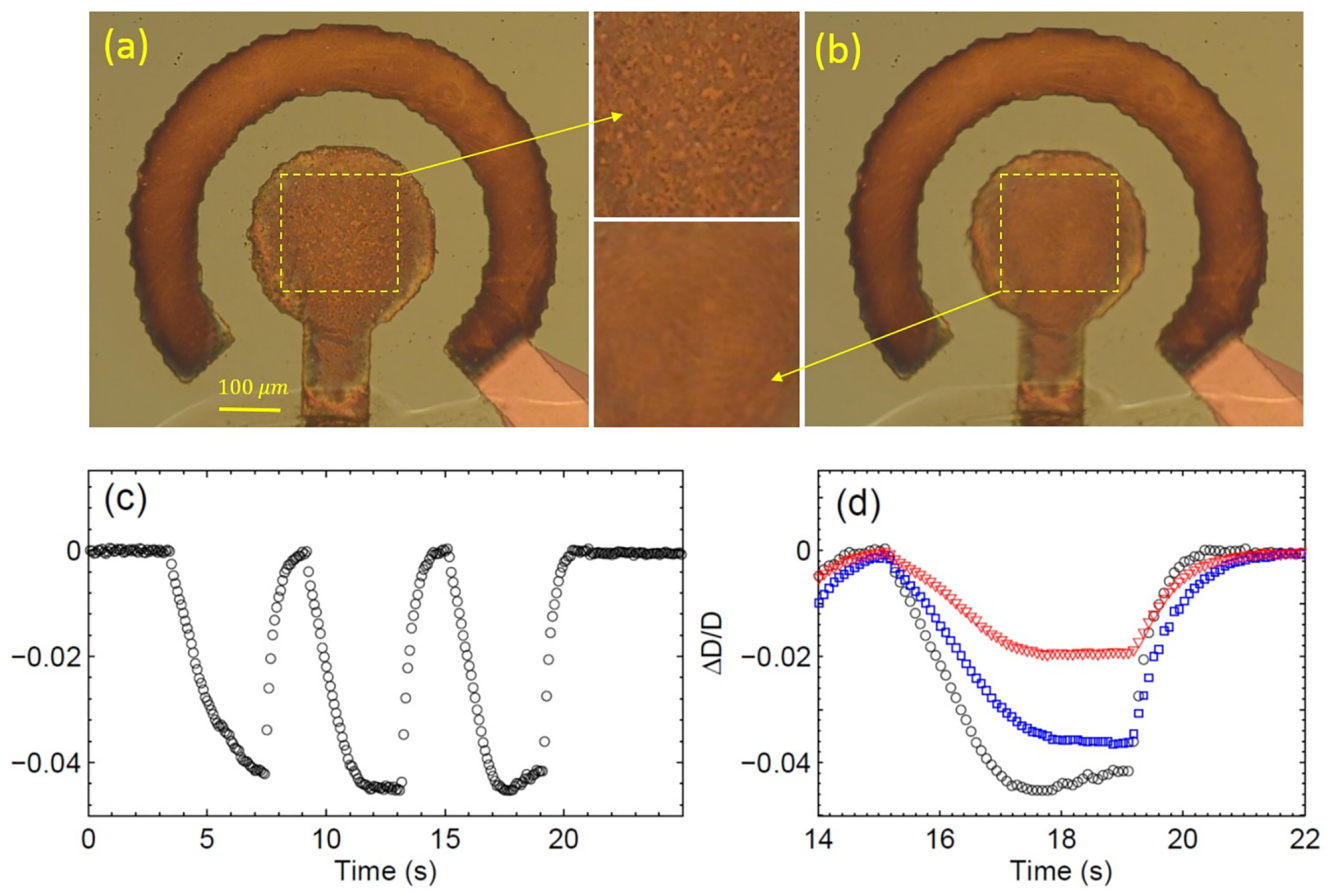
Distortion of the electrodes produced by three series of pulses at *U* = 10 V and *f* = 200 kHz. (a) A snapshot of the electrodes in the very beginning of the third series at *t* = 15 s. (b) The same electrodes 2.6 s later. The central areas within the dashed squares in (a) and (b) are shown as insets in between the panels. (c) The relative change Δ*D*/*D* in the diameter of the central electrode as a function of time. (d) The same change but only for the third series of pulses (starts at *t* = 15 s, ends at *t* = 19 s). The internal circle (electrode) is shown by the black open circles, the middle circle is presented by the blue squares, and the external circle (on the external electrode) is shown by the red triangles.

S1 Video demonstrates the change in the optical image of the electrodes when three series of AP pulses shown in Fig 2 are applied to the electrodes. No visible bubbles are produced during the experiment (each series is 4 s long) but we can observe the distortion of the electrode images. Fig 3(a) is a snapshot of the electrodes in the very beginning of the third series at *t* = 15 *s* but panel (b) shows the same electrodes at *t* = 17.6 *s*. First, one can notice that the diameter of the central electrode is slightly smaller in panel (b). The variation of the diameter is especially well visible in the video. Second, the central electrode in (b) is out of focus as one can see in the insets between the panels. Fig 3(c) shows the relative change of the diameter *D* of the central electrode Δ*D*/*D* with time. Each series of pulses results in the decrease of the visible electrode size up to 4.5% and when the series is over the size gets back to its original value. Variation of the size for the third series of pulses is zoomed in panel (d). After 2.5 s the size saturates at some value. After the last pulse the size returns to its original value faster than in 1 s. The circles defining the peripheral electrode also shrink but in less degree than the central electrode as can be seen in panel (d).

The strongest distortion is observed in the center of the structure and it becomes smaller to the periphery. The effect can be explained by the gas generated in the electrochemical process. We can imagine that the gas produced by the process is distributed in a cloud of a lens-like shape: the layer in the center is thicker than the layer at the periphery. The refractive index of this lens is smaller than that for the surrounding liquid. One would see a similar situation examining an object via a plano-concave lens: the imaginary image of the electrodes would look smaller than it is. An alternative explanation could be that the surface of liquid rises up above the electrodes. This possibility can be excluded since we observe the same situation when a glass is floating above the electrodes.

To estimate the amount of gas that can produce this level of distortion let us assume that the gas-enriched layer above the center of the electrode structure has a thickness *h*. From a simple geometrical optics one can deduce the relation between the relative change of the diameter and the refractive index of the layer (see S1 Appendix)

$$\begin{array}{c} \frac{\Delta D}{D}=-\frac{(M+1)h}{l_{1}}(\frac{1}{n}-\frac{1}{n_{s}}), \end{array}$$ (3)

where *l*_1_ ≈ 33.5 mm is the distance between the object and objective and *M* = 10 is the magnification of the objective. To reproduce the observed Δ*D* the thickness of the gas-enriched layer has to be in the interval *h*_*min*_ < *h* < *H*, where *H* is the thickness of the liquid layer above the electrodes and *h*_*min*_ is the smallest possible thickness corresponding to the smallest possible value of *n*. Of course, the lower limit *n* = 1 (pure gas) cannot be reached in the experiment since we do not observe microbubbles as in [34, 35]. The smallest *n* one can estimate as follows. Suppose that the gas in liquid is collected in clusters (nanobubbles) approximately of similar size. Due to surface tension these clusters are spherical. The maximal volume fraction of gas in such a system is given by the fraction of close-packing of equal spheres that is *f*_*g*_ ≈ 0.74. The corresponding minimal value of the refractive index is *n*_*min*_ = 1.09. Using this *n*_*min*_ and the observed value Δ*D*/*D* ≈ 0.045 we find from Eq (3) *h*_*min*_ ≈ 780 *μ*m. As the ultimate upper limit we use *H* < (3 − 0.5 − 0.1) mm that is the maximal liquid layer minus the thickness of the substrate and the sticking tape. It corresponds to the maximal refractive index *n*_*max*_ = 1.25. Thus, according to Eq (2) the volume fraction of gas in the liquid has to be in the range 0.28 < *f*_*g*_ < 0.74.

It is natural to assume that the optical distortion is due to the gas produced by the electrochemical process. However, one could imaging an alternative explanation when the Faraday current heats up the liquid locally that results in the change of the refractive index. Since the conductivity of electrolytes depends linearly on temperature it can be used to estimate the local temperature near the electrodes. The amplitude of the current is related to the temperature increase Δ*T* as *I* = *I*_0_(1 + *α*Δ*T*), where *α* ≈ 0.024 K^−1^ [30] is the thermal coefficient for 1 mol/L solution of Na_2_SO_4_. As one can see from Fig 2 the current increases slightly during 0.3 s after switching on the pulses, but then it reaches a steady state. The local temperature increase is estimated from the inset in Fig 2 as Δ*T* ≈ 6.5° K. This temperature rise is small enough to exclude a significant volume fraction of vapor, which could influence the refractive index. On the other hand, temperature increase of the solution on 6.5° K will reduce the refractive index less than 0.001 [39], while the observed value is more than 0.1.

If we increase the amplitude of the driving voltage above 12 V, clearly audible clicking sounds will appear, which were described earlier [35] for similar circular electrodes but made from copper. In this case the gas fraction in the electrolyte reaches a critical value *f*_*g*_ = 0.74, when many gaseous clusters merge. The stroboscope images [34] or fast camera videos [35] show the formation of microbubbles, which exist very short time (microseconds) and disappear with a significant release of energy. A manifestation of this energy release is the clicking sound.

### Optical lever experiment

The optical lever experiment gives information on the space structure of the gas cloud around the electrodes. It is known that for a nonhomogeneous medium the beam is deflected in the direction of a larger refractive index. In this experiment the laser beam is going parallel to the sample surface at some vertical distance from this surface and the beam scans along *Y* direction (see Fig 1). When the beam crosses the cloud it always deflects outward. The deflection is measured by the four-section PD. The segments denoted as “Up”, “Down”, “Left”, and “Right” correspond to the direction of the beam deflection relative to the undeflected beam. If there is no cloud the beam incident on the center of the PD on the gap between the segments. The center of the electrodes is chosen as the origin of the coordinate system. When the beam crosses the cloud the PD signal emerges sequentially in “Left”, “Up”, and “Right” segments (the beam moves in the positive direction of *Y* axis). In all cases the signal of the PD responsible for the beam defection towards the sample (segment “Down”) does not change and remains within the noise level. Sectors “Left” and “Right” have their maxima at the distances +250 *μ*m and −250 *μ*m from the center, respectively, indicating a finite size of the cloud. We define the edges of the cloud as the distance where the signal of “Left” and “Right” sectors is half of the maximum that is 500–600 *μ*m. Sector “Up” has the maximal signal in the center of the electrode structure. The total lateral size of the cloud at the height *h* = 500 *μ*m is estimated as *L* = 1000–1200 *μ*m that is in agreement with what we observe with the optical distortion method.

**Fig 4:**
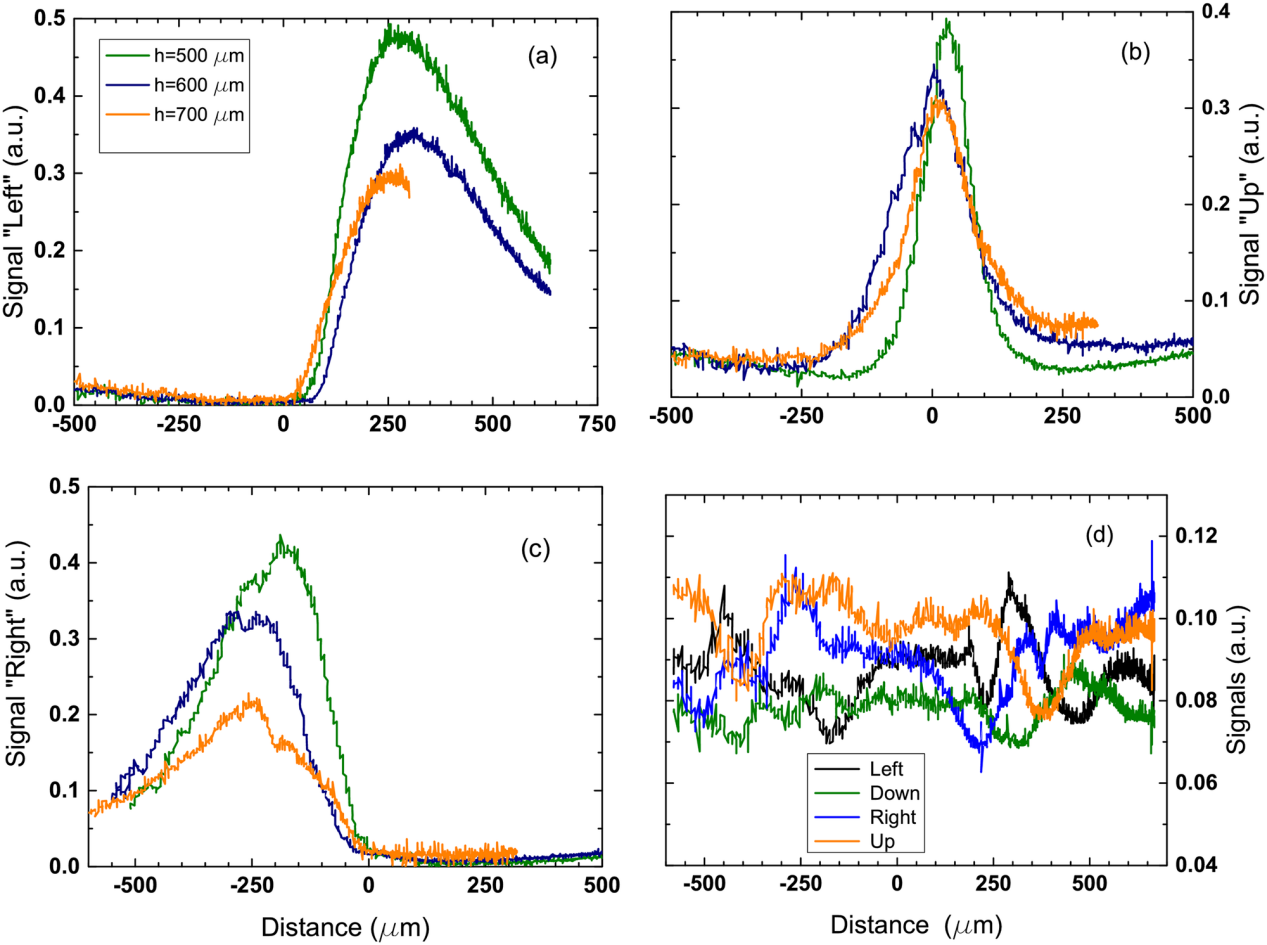
Signal from the PD sections when the laser beam is scanning along *Y* direction (see Fig 1). (a) Signal from “Left” section as a function of *Y*-coordinate. The signal is shown for three different heights (*Z*-coordinates) of the beam above the sample. (b) The signal from “Up” section for the same three heights. (c) The signal from the “Right” section for the same heights. (d) Noise in the system (no pulses applied). The signal from “Down” section is always within the noise.

The deflection data are shown in Fig 4. In panels (a)-(c) the signal is obtained for distances between the beam and substrate equal to *h* = 500, 600, and 700 *μ*m, respectively. One can see that the magnitude of all the signals is slowly reduced with the increasing distance. When the distance is above 1000 *μ*m the signals drops down fast (see S1 Fig). The noise for all the PD segments is shown in panel (d). Of course, representation of the cloud as a spherical cap with a constant concentration of gas as shown in Fig 1 is a simplification but our data show that it can be considered as a reasonable starting guess.

To estimate a homogeneous refractive index *n* of the cloud we present it as a hemispherical one that is not far from what we observe. The radius of this hemisphere is estimated as $R=\sqrt{h^{2}+L^{2}/4}=700-800$ *μ*m for *h* = 500 *μ*m. Using the geometric optics we can relate, for example, the displacement of the laser spot on the “Up” segment of the PD Δ*x* with the change of the refractive index Δ*n* = *n*_*s*_ − *n* as (see S1 Appendix)

$$\begin{array}{c} \Delta n\approx \frac{\Delta xl_{2}\sqrt{1-{\left(h/R\right)}^{2}}}{2l_{1}\left(l-l_{2}\right)\left(h/R\right)}, \end{array}$$ (4)

where *R* is the radius of the cloud, *h* is the vertical distance from the beam to the substrate in the center of the electrode structure, *l*_1_ = 30 mm is the distance between the waist of the beam and the condensing lens, *l*_2_ = 60 mm is the distance between the lens and the image of the waist, and *l* = 210 mm is the distance between the lens and the PD. The displacement Δ*x* is determined from the voltage on the PD as one can see in the calibration curves shown in S2 Fig. For *h* = 500 *μ*m it is Δ*x* ≈ 400 *μ*m. Using the indicated numbers we find Δ*n* ≈ 0.003 that corresponds to the fraction of gas volume in the electrolyte *f*_*g*_ ≈ 0.01. Note that the optical lever method is able to show only excessive gases (above the saturation level) in the liquid. The amount of generated gas is smaller than that in the optical distortion case because much smaller Faraday current *I*_*F*_ ∼ 2 mA is passed through the electrolyte. Small current is necessary to keep the response of the PD linear.

The sensitivity of the system can be estimated from the noise level shown in Fig 4(d). The noise level of a PD segment corresponds to 1 mV that gives a standard deviation in the beam displacement Δ*x* about 40 *μ*m. Using Eq (4) with this Δ*x* one finds for the noise level in *n* a value of *δn* = 3 × 10^−4^.

### DIC experiment

**Fig 5:**
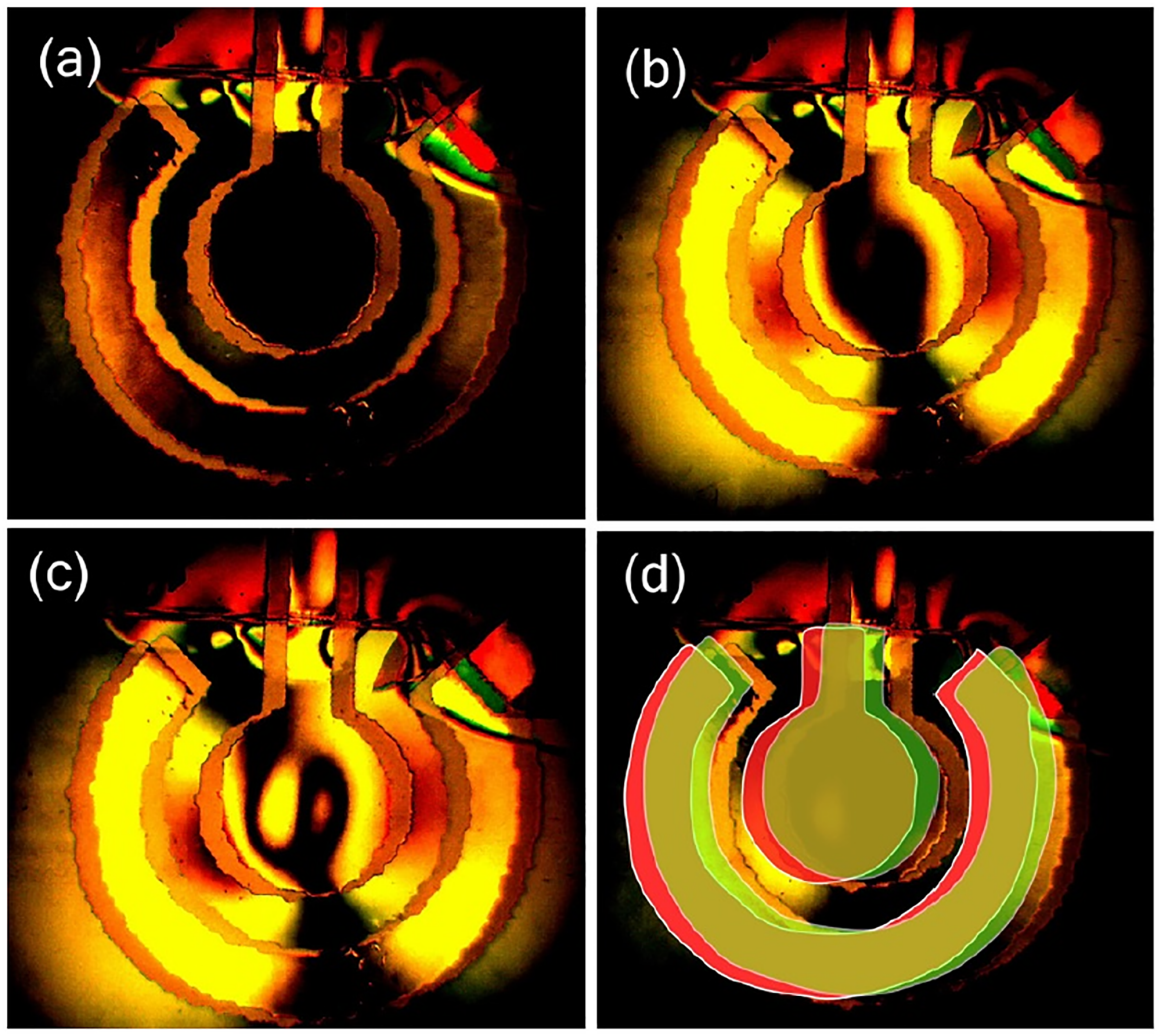
Differential interference contrast in dynamics. (a) Zero amplitude of the driving pulses at *t* = 0. Images for the beams of different polarizations are shifted with respect to each other on 30 *μ*m. (b) Amplitude of the pulses is half of its maximum value at *t* = 2.5 s. (c) Maximal amplitude of the pulses at *t* = 5 s. (d) Scheme of the formation of the DIC.

Change of the differential interference contrast with time due to the pulses modulated by the sawtooth profile is presented in S2 and S3 Videos for two orthogonal directions of the splitting prism. A few frames from S2 Video are shown in Fig 5. One can see two images formed by the Nomarski objective for two different polarizations, which are shifted with respect to each other. In Fig 5(d) the scheme of the contrast formation is presented, where one image is shown red and the other one is shown green. In the overlapping region the interference contrast is high and shown as light-brown. In the regions where there is no overlapping the contrast is low because the substrate is a poor reflector in comparison with the electrodes. Outside of the electrodes the contrast is high again since the interference happens between the beams of similar intensity.

Panel (a) shows the contrast when the amplitude of the pulses is minimal (zero). One can see that in the overlapping regions the phase difference is zero. Some contrast exists only in the upper part of the picture, where the structure is covered by the compound that has a height gradient. Panel (b) shows the contrast when the amplitude of the pulses is half of its maximum value. The central electrode in its center is still black because there is no gradient of the refractive index. However, at the maximal amplitude (panel (c)) some structure appeared in the central part of the electrode that arise due to inhomogeneous current distribution and liquid flow induced by the moving NBs (see below). We observed similar structures in the optical lever data too.

The data demonstrate qualitatively the gas distribution in the cloud that is in agreement with what we observe with the optical distortion and optical lever methods. However, the interpretation of the gradient of *n* is less straightforward and we do not provide here a specific number for *n*.

### Vibrometer experiment

**Fig 6:**
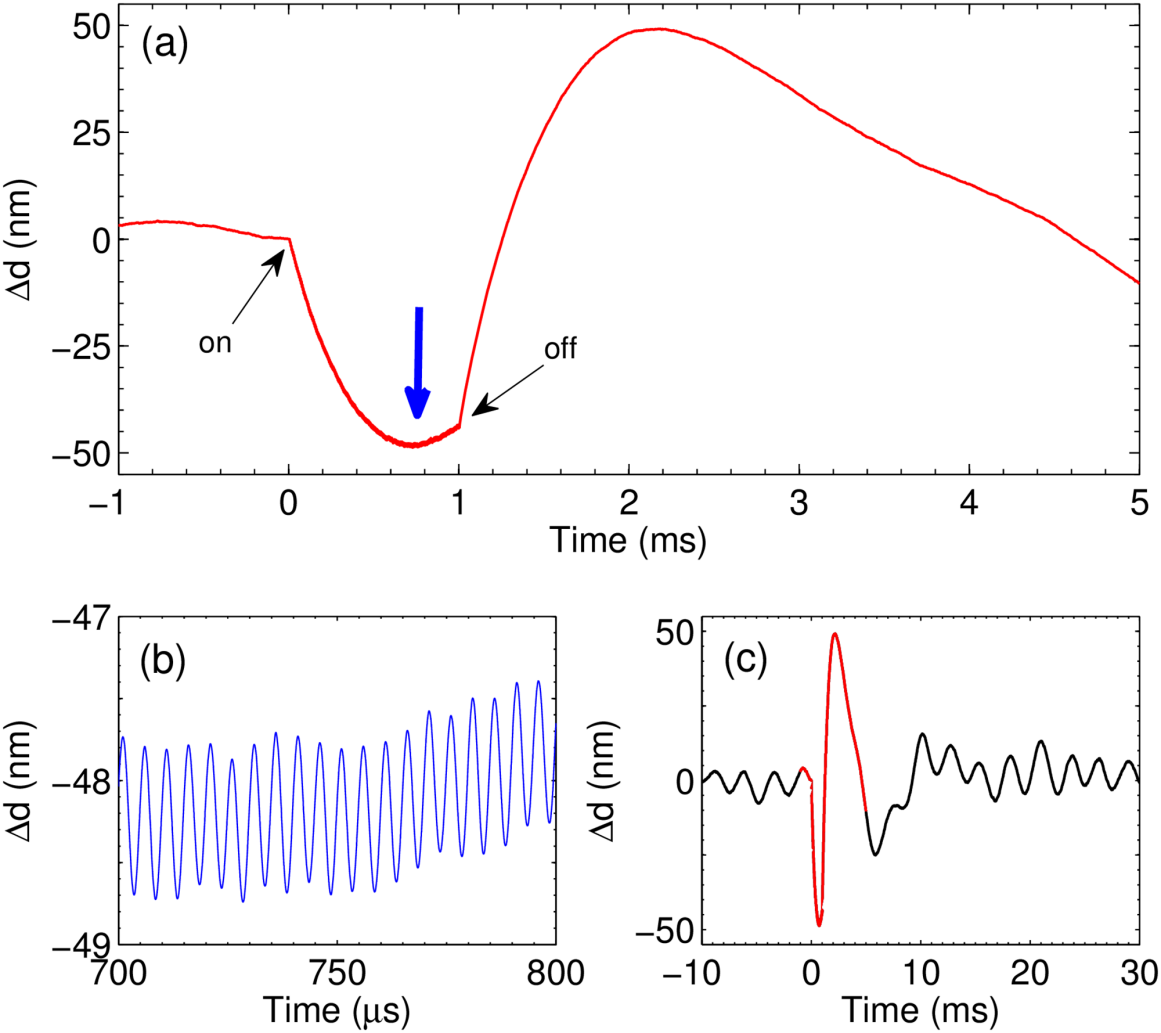
Change of the optical path Δ*d* as measured by the vibrometer. The process is driven by voltage pulses at *f* = 200 kHz. (a) The optical path as a function of time. Moments when the pulses are on and off are indicated. The thick blue arrow shows the place zoomed in panel (b). The signal in (b) is shown in a restricted time domain 0.7 < *t* < 0.8 ms to see oscillations with the driving frequency. (c) The signal in the total time domain. The red part of the curve is the one shown in panel (a).

As we already mentioned the interpretation of the vibrometer data is more complicated because the method is sensitive not only to the change of the refractive index but also to the liquid movement. With this method we can see the change of the refractive index only on a short time scale. Nevertheless, the vibrometer data are important in two aspects. First, the data have a regular character as one can see in Fig 6 demonstrating absence of scattering by objects with a size larger than λ/*π* ≈ 200 nm. Occasionally microscopic bubbles are formed due to presence of defects. They strongly scatter light, and can be easily recognised in the signal as shown in S3 Fig. The second aspect is small oscillations of the signal with the driving frequency *f* = 200 kHz as shown in Fig 6(b). These oscillations are explained by the reaction between hydrogen and oxygen in the NBs containing mixture of gases [29, 30]. Note that the vibrometer signal is completely uncoupled from the driving pulses.

Increase of the gas concentration in the cloud is responsible for smooth evolution of the signal as shown in panel (a). When the pulses are switched on the thickness of the gas-enriched layer increases with time and the signal is negative because the optical path is reduced. New gas clusters produced near the electrodes push the old ones further away generating movement of the liquid. This movement contributes to the signal increasing the optical path. The liquid flow is the reason for the minimum in the signal. When the pulses are switched off the signal evolution is determined by the liquid flow. In the total time domain the signal is shown in Fig 6(c). It demonstrates the noise level, which is dominated by the resonance frequency of the internal mirror of the instrument.

## Discussion

All our observations show that the refractive index changes due to a significant volume fraction of gas, which exists in the electrolyte near the electrodes. We see that the refractive index is reduced that is unambiguously follows from the optical distortion experiment. In this experiment the image of electrodes shrinks in response to the applied AP pulses that is possible only if the refractive index of the cloud is smaller than that for the surrounding liquid. The same is true for the optical lever experiment, for which the beam is always deflected out of the electrode structure.

We cannot determine from our data the chemical nature of the gas that produces significant change of the refractive index. However, there are no doubts that large amount of hydrogen and oxygen is produced by the Faraday current. The total number of molecules *N* produced by one series of pulses in the optical distortion experiment is

$$\begin{array}{c} N=\frac{3I_{F}\tau }{4|e|}\approx 1.1\times 10^{18}, \end{array}$$ (5)

where *I*_*F*_ ≈ 60 mA, *τ* = 4 s, and *e* is the charge of electron. At normal conditions this gas would fill a volume as large as 47 mm^3^. The other gas that could appear in the electrolyte is vapor but the temperature rise is too low to have any appreciable amount of vapor inside of the liquid. No other gases can be produced in the process in appreciable amounts.

The reduction of the refractive index in the distortion experiment was estimated in between 0.1 and 0.26. Any impurity in the solution cannot produce this large change. One could imagine the situation when the amount of salt in the electrolyte varies around the electrodes. However, if the salt concentration drops from 1 mol/L to zero, the refractive index decreases only on 0.02 [40]. Moreover, it would result in a significant reduction of the current, which is not observed. As we already mentioned the local heating also cannot be responsible for the observed change in *n* since the current pulses increase the temperature near the electrodes only on 6.5° K. The only reasonable explanation for the change of *n* in the optical distortion experiment is the gas produced electrochemically.

To answer the question in which form this gas exists in the liquid, let us estimate first the local supersaturation above the electrode. The gas produced by the process diffuses away from the electrode. Within the diffusion layer of thickness $l_{D}∼\sqrt{Dt}$, where *D* is the gas diffusion coefficient and *t* is the time, the gas concentration is estimated as $n_{g}∼\left(j_{F}/e\right)\sqrt{t/D}$, where *j*_*F*_ is the Faraday current density. The latter is *j*_*F*_ ∼ *I*_*F*_/*A* ≈ 85 A/cm^2^, where *A* = 2.25 × 10^−8^ m^2^ is the area of the central electrode. This is an average value of the current density but the local value near the edge of the electrode can be a few times larger [28]. Using as an example the diffusion coefficient for hydrogen *D* = 4.5 × 10^−9^ m^2^/s one finds that just in 50 *μ*s the gas concentration reaches the value *n*_*g*_ ∼ 5.6 × 10^26^ m^−3^, which corresponds to the supersaturation *S* = *n*_*g*_/*n*_*sat*_ ∼ 1200, where the saturated concentration for H_2_ is *n*_*sat*_ = 4.7 × 10^23^ m^−3^.

This huge supersaturation reduces the barrier for bubble nucleation and bubbles are generated homogeneously in the diffusion layer. It was demonstrated [28] that hydrogen produced by a 50 *μ*s long single polarity pulse manifests itself in the form of nanobubbles covering the negative electrode homogeneously already in 20 *μ*s. These NBs scatter light and are visible as a haze. After 100 *μ*s they form well visible microbubbles and their dynamics becomes slow. It is interesting to note that although the NBs cover a significant area of the electrode the current is not reduced. It means that the nucleation most of the bubbles happens above the surface. Indeed, this is possible only due to the homogeneous nucleation when surface defects do not play a crucial role in the nucleation.

A somewhat different scenario is realized for alternating polarity pulses because both gases are produced above the same electrode and these gases are able to interact. When a NB contains mixture of gases they react to each other apparently via surface dominating processes [26, 29, 31]. This effect appears as periodic reduction of the gas concentration in the liquid as demonstrated by the oscillations in the vibrometer signal (see Fig 6(b)). If the mixture of gases in a bubble is not stoichiometric, this bubble ends up as a pure hydrogen or a pure oxygen bubble. Such NBs containing only H_2_ or only O_2_ are collected in the system and are responsible for the change of the refractive index in our experiments. Therefore, we conclude that the gas in the liquid exists in the form of NBs and the main reason for this is extremely high supersaturation produced by the electrochemical process in AP regime.

The next question is why the NBs are not grow to the size strongly scattering the visible light? Two bubbles containing different gases can exchange by the content due to gas diffusion in the liquid (Ostwald ripening). In this process one bubble disappears at all but the other one is not growing because the gas that enters in the bubble disappears in the reaction. If the pulses are switched off and the gas is not produced, this effect reduces the total gas concentration as one can see in Fig 3(d). While the pulses are switched on, the dynamical equilibrium is reached, which is responsible for a plateau in Fig 3(d). The same equilibrium provides the hemispherical shape of the cloud instead of a cylindrical shape. At some distance from the electrodes nearly all the gas is reacted and such a state is supported by the produced gas.

The discussion above shows that it is hardly possible to visualize individual NBs with the existing methods because they are not only very small but also live a short time. These NBs probably are not different from the bulk NBs produced mechanically [16, 17], but their short lifetime is related to the possibility of the gases to react. Although we observe collective effect and do not see individual NBs, we believe that it is possible to conclude that the cloud observed optically consists of the bulk NBs with the size smaller than 200 nm. The reason for this believe is that the gas has to cluster in 10 *μ*s or less for supersaturations ∼ 1000 reached in the AP electrochemical process.

Nanobubbles open the possibility for a high effective supersaturation of liquids. Since the gas in NBs is densely packed due to a high Laplace pressure, liquid can accept much more gas than in the form of microscopic bubbles where the Laplace pressure does not dominate. We can estimate the level of effective supersaturation in the cloud using the result of the distortion experiment. Using the definition of the volume gas fraction *f*_*g*_ and the ideal gas law for hydrogen concentration one has

$$\begin{array}{c} n_{H_{2}}=\frac{2f_{g}\left(P_{0}+2\gamma /r\right)}{3kT}, \end{array}$$ (6)

where *r* is the bubble size, *γ* is the surface tension of the liquid, *P*_0_ is the atmospheric pressure, and *kT* is the temperature in energy units. Taking as reasonable values *f*_*g*_ = 0.5 and *r* = 50 nm one finds *n*_*H*_2__ ≈ 2.3 × 10^26^ m^−3^, which corresponds to the supersaturation with hydrogen *S*_*H*_2__ ≈ 495. The concentration of oxygen is two times smaller than *n*_*H*_2__ and the supersaturation is *S*_*O*_2__ ≈ 152 (saturated concentration for O_2_ is 7.7 × 10^23^ m^−3^). These values of *S* are smaller than those achieved near the electrodes because significant part of the produced gas turns back to water, but anyway, the level of supersaturation in the cloud is very high.

If *V* is the volume of one NB, then the average volume per one bubble in the cloud is *V*/*f*_*g*_. For the optical distortion experiment 0.28 < *f*_*g*_ < 0.74 and one can say that the state of the material is rather a nanofoam than a liquid. This is the reason why the refractive index strongly deviates from that of liquid. With the increase of the voltage amplitude the Faraday current becomes larger and the volume fraction of gas increases. At *U* = 12 V this fraction apparently reaches its maximal value *f*_*g*_ = 0.74 because short-lived microbubbles start to appear in the system, which explode with well audible sound. This effect was described earlier [35] for the electrodes of a similar geometrical shape but made from copper and having a slightly larger size. The appearance of short-lived microbubbles was explained there by coalescence of many NBs due to their dense packing. The present work supports this conclusion.

In the optical lever experiment the Faraday current *I*_*F*_ ≈ 2 mA was much smaller than in the optical distortion experiment, where *I*_*F*_ ≈ 60 mA. In the former case we have found much smaller gas fraction *f*_*g*_ ≈ 0.01 than in the latter one. It clearly demonstrates that the density of NBs in the cloud is defined by the Faraday current as one could expect for an electrochemical process.

## Conclusions

In this paper we visualized a cloud of NBs using different experimental techniques. The most important property of the cloud is that in spite of a significant gas content it does not scatter visible light demonstrating absence of objects with a size larger than λ/*π*, where λ = 600 nm is a typical wavelength of light. At the same time it has a significantly different refractive index, which is smaller than the index of the surrounding liquid. The cloud can be roughly represented as a hemispherical segment based on the electrodes and having the diameter two times larger than the external diameter of the electrodes.

When the voltage pulses are applied to the electrodes the cloud is growing with a rate defined by the electrochemical current but then it reaches a steady state. The latter is explained as a balance between new bubbles produced by the current and the bubbles merging and disappearing in the combustion reaction. When the pulses are switched off the cloud disappears for less than a second. This process is controlled only by merging of the bubbles.

Here we demonstrated that the cloud of bulk NBs can be very dense and it can be controlled electrically. The density of the cloud is defined by the Faraday current. It can be important for applications ranging from cleaning to applications in microfluidic devices.

## Supporting information

S1 Fig Upper boundary of the cloud. Signal of “Up” segment as a function of *Y*-coordinate for four different beam distances from the substrate. The signal is going down fast when the vertical distance is larger than 800 *μ*m.
(JPG)

S2 Fig Calibration curves. Signal on the PD segments “Up” and “Down” versus the position of the laser spot on the PD surface. Coordinate “0” corresponds to the center of the PD. The curves are used as the calibration curves.
(JPG)

S3 Fig Scattering on microbubbles. Raw signal of the vibrometer (velocity) for a very high driving voltage *U* = 18 V averaged over a period of 5 *μ*s. The left panel (a) shows the response to 2500 pulses triggered at *t* = 0. The signal jumps up and down in the beginning and at the end of the series. No scattering on microbubbles is observed. Similar raw signal but of smaller magnitude, corresponds to Fig 6 in the main text. The right panel (b) shows the signal for a longer series of 4000 pulses. Distinctive spikes appear due to scattering on microbubbles, two of them are indicated by the arrows.
(JPG)

S1 Video Optical distorsion of the electrodes. Video was recorded at *U* = 10 V and *f* = 200 kHz without a glass floating above the electrodes. The frame rate is 60 fps and the frame size is 1920 × 1080 px. The video with a glass floating above the electrodes is looking very similar and is available on the request. The corresponding voltage and current are shown in Fig 2 in the main text.
(AVI)

S2 Video Differential interference contrast of the cloud. File was recorded at *U* = 8 V and *f* = 400 kHz at a frame rate of 30 fps. The frame size is 1280 × 1024 px. The voltage is modulated by the sawtooth profile with a period of 5 s.
(AVI)

S3 Video Differential interference contrast, orthogonal polarization. The same as S2 Video but the splitting prism was rotated 90° in comparison with S2 Video. The color gradient from green to red is due to small inclination of the sample.
(AVI)

S1 Appendix Derivation of Eqs (3) and (4). (PDF)

## References

[1] Seddon JRT, Lohse D, Ducker WA, Craig VSJ. A deliberation on nanobubbles at surfaces and in bulk. Chem Phys Chem. 2012;13(8):2179–2187. 10.1002/cphc.201100900 22378608

[2] Lohse D, Zhang X. Surface nanobubbles and nanodroplets. Rev Mod Phys. 2015;87:981–1035. 10.1103/RevModPhys.87.981

[3] Alheshibri M, Qian J, Jehannin M, Craig VSJ. A History of Nanobubbles. Langmuir. 2016;32(43):11086–11100. 10.1021/acs.langmuir.6b02489 27594543

[4] Wu Z, Chen H, Dong Y, Mao H, Sun J, Chen S, et al Cleaning using nanobubbles: Defouling by electrochemical generation of bubbles. J Colloid Interface Sci. 2008;328(1):10–14. 10.1016/j.jcis.2008.08.064 18829043

[5] Liu G, Wu Z, Craig VSJ. Cleaning of Protein-Coated Surfaces Using Nanobubbles: An Investigation Using a Quartz Crystal Microbalance. J Phys Chem C. 2008;112(43):16748–16753. 10.1021/jp805143c

[6] Liu G, Craig VSJ. Improved Cleaning of Hydrophilic Protein-Coated Surfaces using the Combination of Nanobubbles and SDS. ACS Appl Mater Interfaces. 2009;1(2):481–487. 10.1021/am800150p 20353240

[7] Zhu J, An H, Alheshibri M, Liu L, Terpstra PMJ, Liu G, et al Cleaning with Bulk Nanobubbles. Langmuir. 2016;32(43):11203–11211. 10.1021/acs.langmuir.6b01004 27109142

[8] Wang Y, Bhushan B, Zhao X. Improved Nanobubble Immobility Induced by Surface Structures on Hydrophobic Surfaces. Langmuir. 2009;25(16):9328–9336. 10.1021/la901186a 19572534

[9] Wang Y, Bhushan B. Boundary slip and nanobubble study in micro/nanofluidics using atomic force microscopy. Soft Matter. 2010;6:29–66. 10.1039/B917017K

[10] Tasaki T, Wada T, Baba Y, Kukizaki M. Degradation of Surfactants by an Integrated Nanobubbles/VUV Irradiation Technique. Ind Eng Chem Res. 2009;48(9):4237–4244. 10.1021/ie801279b

[11] Agarwal A, Ng WJ, Liu Y. Principle and applications of microbubble and nanobubble technology for water treatment. Chemosphere. 2011;84(9):1175–1180. 10.1016/j.chemosphere.2011.05.054 21689840

[12] Mishchuk N, Ralston J, Fornasiero D. Influence of very small bubbles on particle/bubble heterocoagulation. J Colloid Interface Sci. 2006;301(1):168–175. 10.1016/j.jcis.2006.04.071 16725149

[13] Azevedo A, Etchepare R, Calgaroto S, Rubio J. Aqueous dispersions of nanobubbles: Generation, properties and features. Miner Eng. 2016;94:29–37. 10.1016/j.mineng.2016.05.001

[14] Mondal S, Martinson JA, Ghosh S, Watson R, Pahan K. Protection of Tregs, Suppression of Th1 and Th17 Cells, and Amelioration of Experimental Allergic Encephalomyelitis by a Physically-Modified Saline. PLoS One. 2012;7(12):e51869 10.1371/journal.pone.0051869 23284794 PMC3527485

[15] Modi KK, Jana A, Ghosh S, Watson R, Pahan K. A Physically-Modified Saline Suppresses Neuronal Apoptosis, Attenuates Tau Phosphorylation and Protects Memory in an Animal Model of Alzheimer’s Disease. PLoS One. 2014;9(8):e103606 10.1371/journal.pone.0103606 25089827 PMC4121132

[16] Ohgaki K, Khanh NQ, Joden Y, Tsuji A, Nakagawa T. Physicochemical approach to nanobubble solutions. Chem Eng Sci. 2010;65(3):1296–1300. 10.1016/j.ces.2009.10.003

[17] Ushikubo FY, Furukawa T, Nakagawa R, Enari M, Makino Y, Kawagoe Y, et al Evidence of the existence and the stability of nano-bubbles in water. Colloids Surf A: Physicochem Eng Asp. 2010;361(1–3):31–37. 10.1016/j.colsurfa.2010.03.005

[18] Bunkin NF, Shkirin AV, Ignatiev PS, Chaikov LL, Burkhanov IS, Starosvetskij AV. Nanobubble clusters of dissolved gas in aqueous solutions of electrolyte. I. Experimental proof. J Chem Phys. 2012;137(5). 10.1063/1.4739528 22894370

[19] Yurchenko SO, Shkirin AV, Ninham BW, Sychev AA, Babenko VA, Penkov NV, et al Ion-Specific and Thermal Effects in the Stabilization of the Gas Nanobubble Phase in Bulk Aqueous Electrolyte Solutions. Langmuir. 2016;32(43):11245–11255. 10.1021/acs.langmuir.6b01644 27350310

[20] Kikuchi K, Takeda H, Rabolt B, Okaya T, Ogumi Z, Saihara Y, et al Hydrogen particles and supersaturation in alkaline water from an Alkali–Ion–Water electrolyzer. J Electroanal Chem. 2001;506(1):22–27. 10.1016/S0022-0728(01)00517-4

[21] Kikuchi K, Tanaka Y, Saihara Y, Ogumi Z. Study of hydrogen nanobubbles in solution in the vicinity of a platinum wire electrode using double-potential step chronoamperometry. Electrochim Acta. 2006;52(3):904–913. 10.1016/j.electacta.2006.06.026

[22] Kikuchi K, Tanaka Y, Saihara Y, Maeda M, Kawamura M, Ogumi Z. Concentration of hydrogen nanobubbles in electrolyzed water. J Colloid Interface Sci. 2006;298(2):914–919. 10.1016/j.jcis.2006.01.010 16445932

[23] Kikuchi K, Nagata S, Tanaka Y, Saihara Y, Ogumi Z. Characteristics of hydrogen nanobubbles in solutions obtained with water electrolysis. J Electroanal Chem. 2007;600(2):303–310. 10.1016/j.jelechem.2006.10.005

[24] Kikuchi K, Ioka A, Oku T, Tanaka Y, Saihara Y, Ogumi Z. Concentration determination of oxygen nanobubbles in electrolyzed water. J Colloid Interface Sci. 2009;329(2):306–309. 10.1016/j.jcis.2008.10.009 18977493

[25] Pan G, He G, Zhang M, Zhou Q, Tyliszczak T, Tai R, et al Nanobubbles at Hydrophilic Particle–Water Interfaces. Langmuir. 2016;32(43):11133–11137. 10.1021/acs.langmuir.6b01483 27180638

[26] Svetovoy V, Postnikov A, Uvarov I, Sanders R, Krijnen G. Overcoming the Fundamental Limit: Combustion of a Hydrogen-Oxygen Mixture in Micro- and Nano-Bubbles. Energies. 2016;9(2):94 10.3390/en9020094

[27] Vogt H, Balzer RJ. The bubble coverage of gas-evolving electrodes in stagnant electrolytes. Electrochimica Acta. 2005;50(10):2073–2079. 10.1016/j.electacta.2004.09.025

[28] Svetovoy VB, Sanders RGP, Elwenspoek MC. Transient nanobubbles in short-time electrolysis. J Phys: Cond Matter. 2013;25(18):184002. 10.1088/0953-8984/25/18/184002 23598648

[29] Svetovoy VB, Sanders RGP, Lammerink TSJ, Elwenspoek MC. Combustion of hydrogen-oxygen mixture in electrochemically generated nanobubbles. Phys Rev E. 2011;84:035302(R) 10.1103/PhysRevE.84.035302 22060445

[30] Svetovoy VB, Sanders RGP, Ma K, Elwenspoek MC. New type of microengine using internal combustion of hydrogen and oxygen. Sci Rep. 2014;4:4296 10.1038/srep04296 24599052 PMC3944672

[31] Prokaznikov A, Tas N, Svetovoy V. Surface Assisted Combustion of Hydrogen-Oxygen Mixture in Nanobubbles Produced by Electrolysis. Energies. 2017;10(2):178 10.3390/en10020178

[32] Takahashi M, Chiba K, Li P. Free-Radical Generation from Collapsing Microbubbles in the Absence of a Dynamic Stimulus. J Phys Chem B. 2007;111(6):1343–1347. 10.1021/jp0669254 17253740

[33] Li P, Takahashi M, Chiba K. Enhanced free-radical generation by shrinking microbubbles using a copper catalyst. Chemosphere. 2009;77(8):1157–1160. 10.1016/j.chemosphere.2009.07.062 19781738

[34] Postnikov AV, Uvarov IV, Prokaznikov AV, Svetovoy VB. Observation of spontaneous combustion of hydrogen and oxygen in microbubbles. Appl Phys Lett. 2016;108:121604 10.1063/1.4944780

[35] Postnikov AV, Uvarov IV, Lokhanin MV, Svetovoy VB. Highly energetic phenomena in water electrolysis. Sci Rep. 2016;6:39381 10.1038/srep39381 27982103 PMC5159792

[36] Murphy DP. Fundamentals of Light Microscopy and Electronic Imaging. New York: Wiley-Liss; 2001.

[37] Gils SV, Mast P, Stijns E, Terryn H. Colour properties of barrier anodic oxide films on aluminium and titanium studied with total reflectance and spectroscopic ellipsometry. Surf Coat Technol. 2004;185(2–3):303–310. 10.1016/j.surfcoat.2004.01.021

[38] Aspnes DE. Optical properties of thin films. Thin Solid Films. 1982;89(3):249–262. 10.1016/0040-6090(82)90590-9

[39] Thormaehlen I, Straub J, Grigull U. Refractive Index of Water and Its Dependence on Wavelength, Temperature, and Density. J Phys Chem Ref Data. 1985;14(4).

[40] Urréjola S, Sánchez A, Hervello MF. Refractive Indices of Sodium, Potassium, and Ammonium Sulfates in Ethanol–Water Solutions. J Chem Eng Data. 2010;55(8):2924–2929. 10.1021/je9010129

