# Supplementary figures and images for: Electrically controlled cloud of bulk nanobubbles in water solutions
Source: PLoS One. 2017 Jul 20;12(7):e0181727. doi: 10.1371/journal.pone.0181727 (PMC5519201; doi:10.1371/journal.pone.0181727)

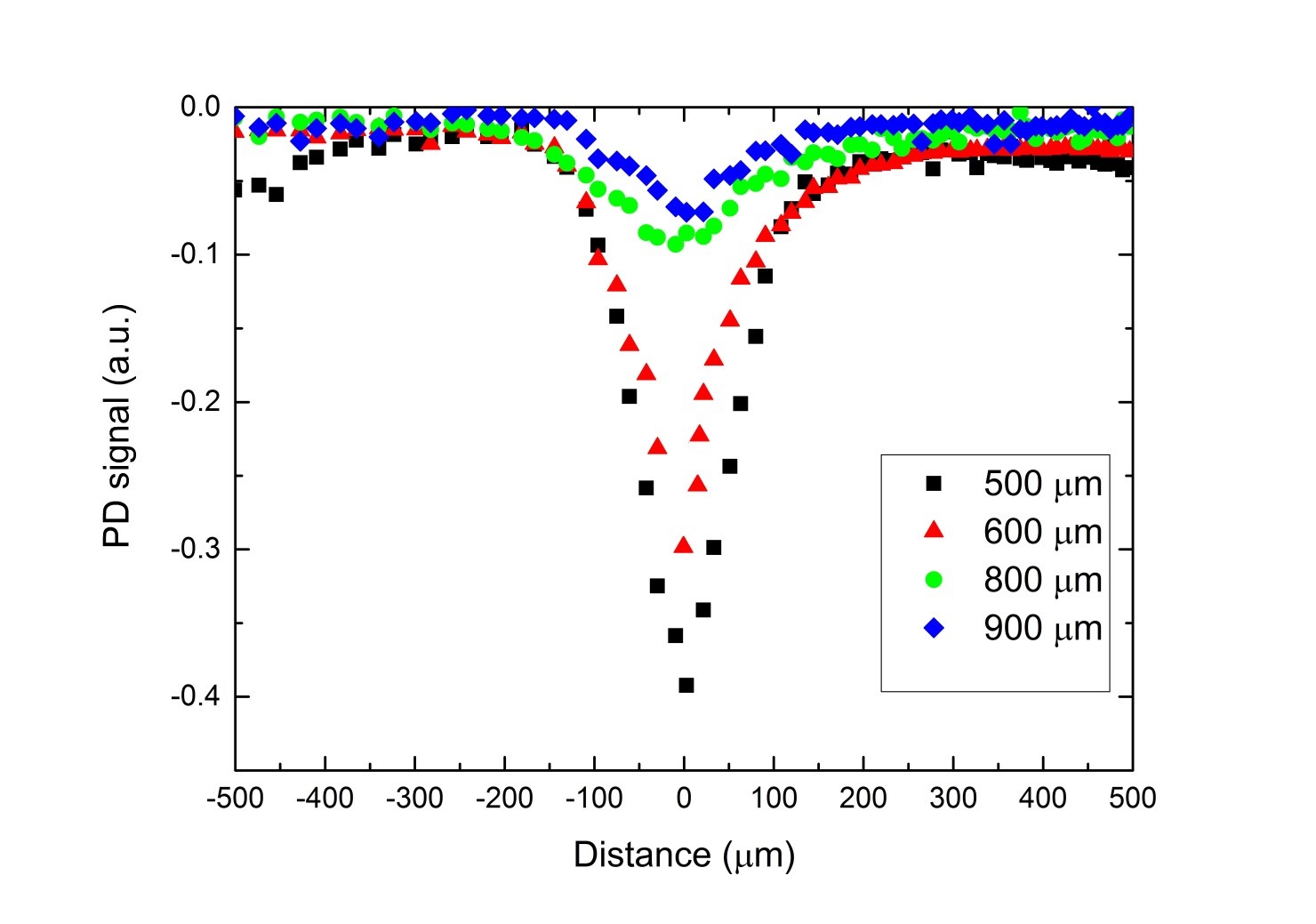

Supplement: S1 Fig — Signal of “Up” segment as a function of Y-coordinate for four different beam distances from the substrate. The signal is going down fast when the vertical distance is larger than 800 μm. (JPG) [file pone.0181727.s001.jpg]

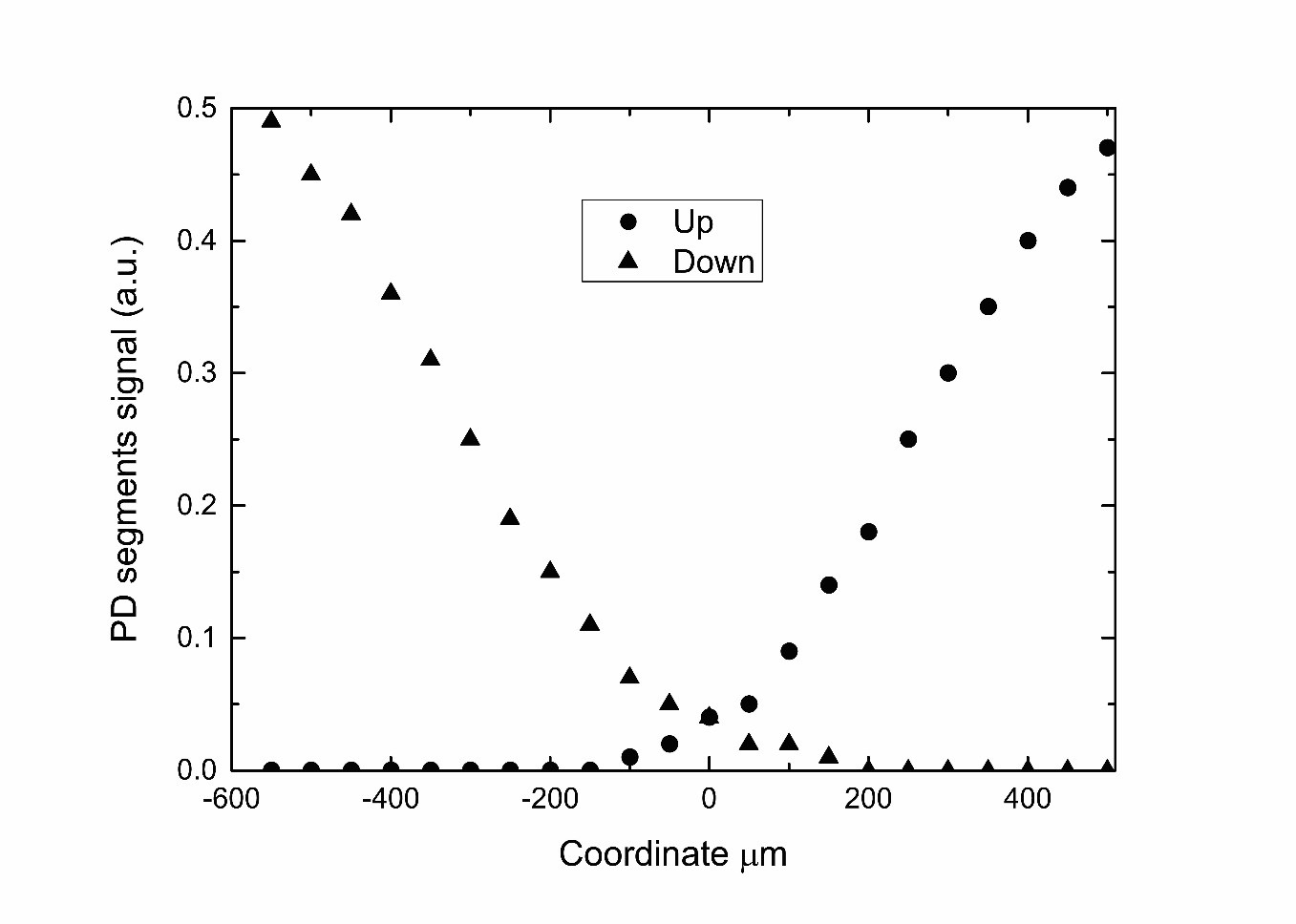

Supplement: S2 Fig — Signal on the PD segments “Up” and “Down” versus the position of the laser spot on the PD surface. Coordinate “0” corresponds to the center of the PD. The curves are used as the calibration curves. (JPG) [file pone.0181727.s002.jpg]

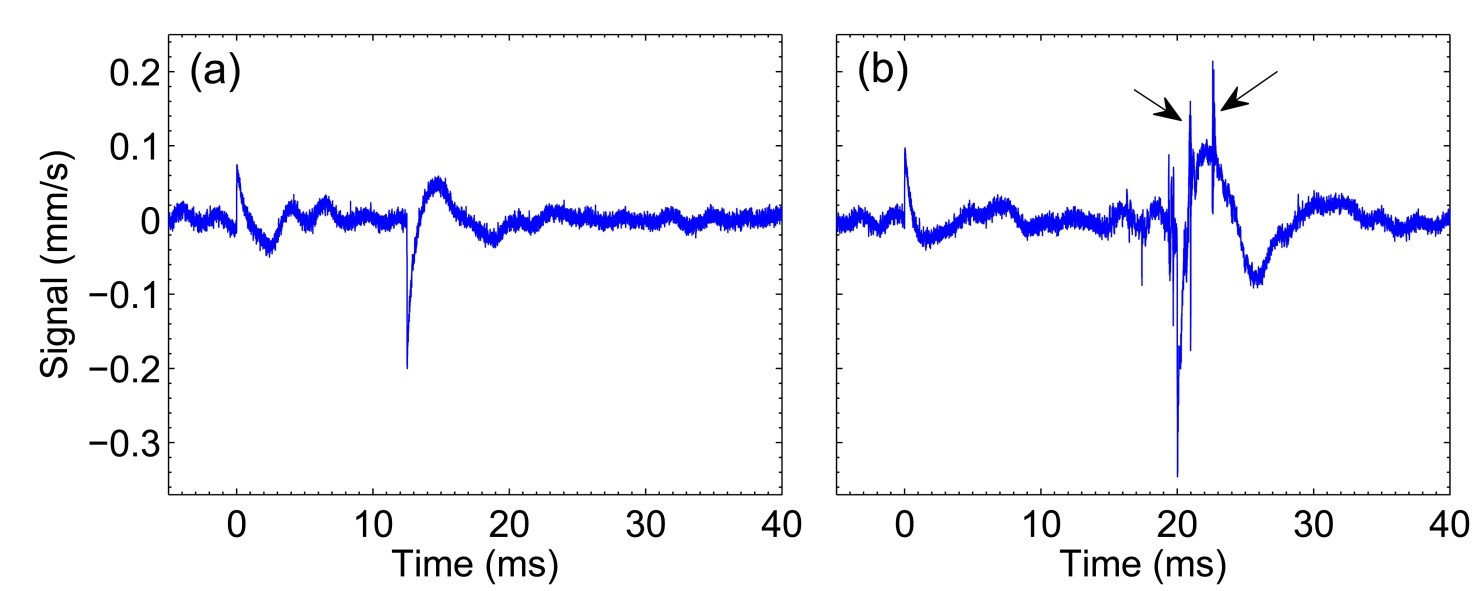

Supplement: S3 Fig — Raw signal of the vibrometer (velocity) for a very high driving voltage U = 18 V averaged over a period of 5 μs. The left panel (a) shows the response to 2500 pulses triggered at t = 0. The signal jumps up and down in the beginning and at the end of the series. No scattering on microbubbles is observed. Similar raw signal but of smaller magnitude, corresponds to Fig 6 in the main text. The right panel (b) shows the signal for a longer series of 4000 pulses. Distinctive spikes appear due to scattering on microbubbles, two of them are indicated by the arrows. (JPG) [file pone.0181727.s003.jpg]
