# Supplementary material for: Electrically controlled cloud of bulk nanobubbles in water solutions
Source: PLoS One. 2017 Jul 20;12(7):e0181727. doi: 10.1371/journal.pone.0181727 (PMC5519201; doi:10.1371/journal.pone.0181727)
Supplement: S1 Appendix — (PDF) [file pone.0181727.s007.pdf]

Appendix

Equation (3) in the main text follows from simple geometric optics. The liquid layer above the central electrode consists of a layer with a thickness h_1, which is enriched with gas and has a refractive index n_1, and a layer of the solution with a thickness h_2 and refractive index n_2 as shown in Fig. 5S. The gas above the liquid layer has the refractive index $n_0 = 1$.

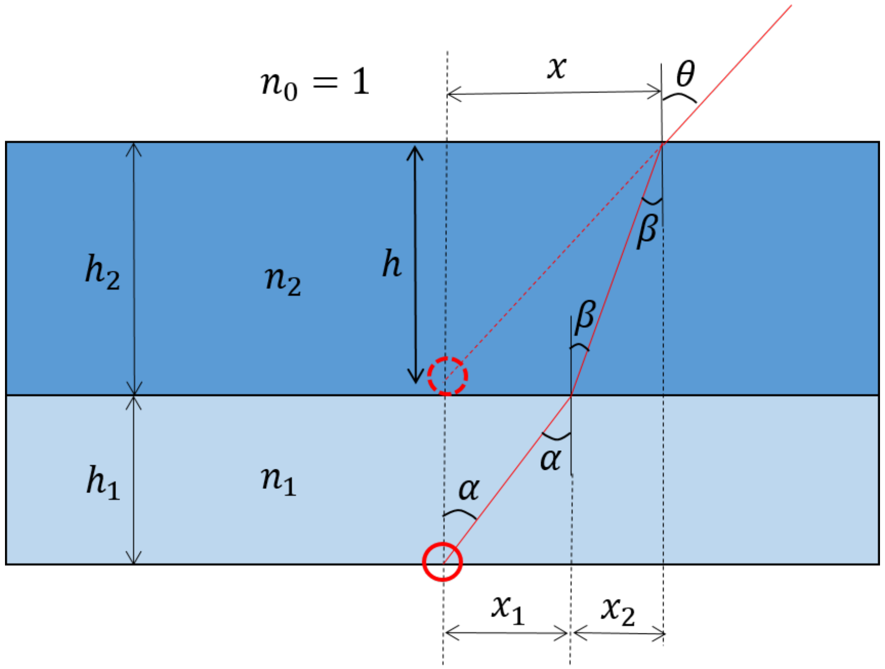

Figure 1A. Image of a point in the center of electrodes (in red circle) for two layer structure. The imaginary image of the point is in the dashed red circle at a depth h. The light rays are shown by the red lines.

The distance $x = x_1 + x_2$ is expressed from Fig. 4S as

$$h \tan \theta = h_1 \tan \alpha + h_2 \tan \beta.$$

The angles α, β and θ are related by the Snell law and for small angles one finds

$$h = h_1 \left(\frac{1}{n_1} - \frac{1}{n_2} \right) + \frac{h_1 + h_2}{n_2}.$$

The change in the position of the imaginary image Δh due to presence of the gas enriched layer is defined only by the first term

$$\Delta h = h_1 \left(\frac{1}{n_1} - \frac{1}{n_2} \right). \quad (1)$$

If we examine an object with the size D_o via an imaging lens, its image size D_i is related to the to the distances from the object to the microscope lens l_1 and from the lens to the image l_2 as

$$\frac{D_i}{D_0} = \frac{l_2}{l_1}. \tag{2}$$

Due to presence of the gas enriched layer the distance l_1 is augmented by Δh. The distance l_2 also changes since it is related to l_1 by the lens equation. Differentiating Eq. (2) one can find the relative change of the image size related to the variation of l_1 as

$$\frac{\Delta D_i}{D_i} = -(M+1)\frac{\Delta h}{l_1}, \tag{3}$$

where $M = l_2/l_1$ is the lens magnification. This relation coincides with Eq. (3) in the main text, where the sign is omitted.

Derivation of Eq. (4) in the main text is also based on geometric optics. A cross-section of the cuvette is shown in Fig. 5S(a). The cloud of nanobubbles is presented as a hemisphere of radius R. A light ray enters the cuvette parallel to its bottom at a height h above the sample, passes through the cloud with two refractions, and exits from the cuvette at the angle θ with an additional refraction.

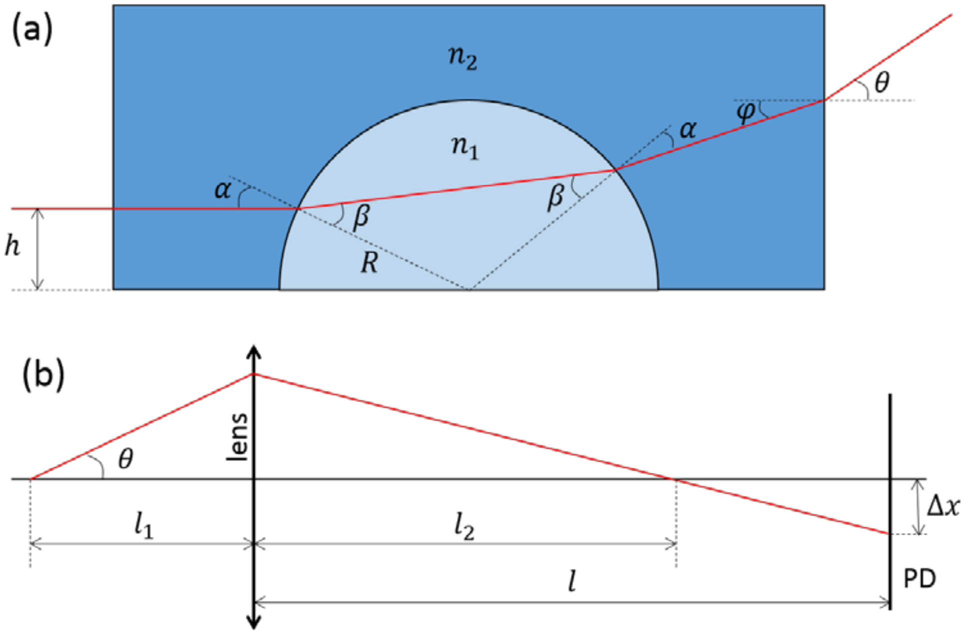

Figure 2A. (a) A ray passing through the cuvette with the hemispherical cloud of nanobubbles is shown by the red line. Important angles are indicated in the figure. (b) The ray that exits the cuvette is collected by the lens and projected to the photodiode (PD). Displacement of the ray Δx on the PD can be expressed via the geometric characteristics shown in the figure.

As follows from geometry $\varphi = 2(\beta - \alpha)$ and $\sin\alpha = h/R$. The angles β and θ can be expressed via Snell's law as

$$\sin\beta = \frac{n_2}{n_1}\sin\alpha, \quad \sin\theta = n_2 \sin 2(\beta - \alpha).$$

Since the difference of the refractive indexes is small $\Delta n = n_2 - n_1 \ll n_2$ the angle β is close to α and θ is small. In this approximation one finds

$$\theta \approx 2\Delta n \tan\alpha. \tag{4}$$

The laser beam that exits the cuvette is collected by the lens and projected to the photodiode. The displacement of the beam position Δx can be expressed using Fig. 5(b) as

$$\Delta x = \frac{l - l_2}{l_2} l_1 \tan\theta. \tag{5}$$

Combining (4) and (5) one finds for Δn

$$\Delta n \approx \frac{\Delta x l_2}{2 l_1 (l - l_2)} \cot\alpha. \tag{6}$$

It coincides with Eq. (4) in the main text if we express $\cot\alpha$ via the ratio h/R.
